# Access to Care for Homeless Veterans During Disasters

**DOI:** 10.1177/2150132718815382

**Published:** 2018-12-04

**Authors:** Alicia R. Gable, Claudia Der-Martirosian, Aram Dobalian

**Affiliations:** 1 Veterans Emergency Management Evaluation Center (VEMEC), US Department of Veterans Affairs, North Hills, CA, USA; 2 University of Memphis School of Public Health, Memphis, TN, USA

**Keywords:** disaster, homeless, access to care, veterans, Medicaid

## Abstract

**Introduction:** Since 1970, natural disasters have led to both temporary and permanent closures of multiple medical centers and outpatient clinics at the US Department of Veterans Affairs (VA) nationwide. Access to care during such events is critical for vulnerable populations, especially homeless veterans. As such, facility closures may disproportionately affect homeless veteran patients who are both more likely to experience adverse effects from disasters and face multiple barriers to care. **Methods:** A cross-sectional survey was administered to a probability sample of 2000 homeless VA patients living in and receiving VA health care in the Northeast United States. The survey was completed by 383 respondents (20% adjusted response rate). This pilot study examines predictors of difficulty accessing care in the event that the VA facility that homeless VA patients routinely use is forced to close because of a natural disaster. **Results:** In a multivariate logistic regression, homeless VA patients who had Medicaid were less likely (OR 0.38; 95% CI: 0.18-0.78; *P* < .01) to report that they would have difficulty obtaining care elsewhere if their normal VA facility was closed in a future natural disaster. **Conclusions:** Findings suggest that Medicaid coverage has the potential to facilitate access to care for homeless veteran VA patients during disasters. Policy changes that decrease Medicaid coverage could limit access to care for homeless veterans during closures of VA medical facilities.

## Introduction

The US Department of Veterans Affairs (VA) serves an important safety net function for veterans experiencing and at risk for homelessness. A 2015 national survey estimated that 1 in 12 veterans experiences homelessness (lacking permanent housing and living in a shelter, transitional housing, outdoors, or other unstable situation) during their lifetime.^1^ Between 2006 and 2013, 599 872 (approximately 10%) veterans enrolled in VA health care had experienced or were at risk of homelessness.^2^ VA has invested heavily in efforts to end veteran homelessness^3^ and provides housing, health care, job training, and social support services to homeless and at-risk veterans.^4^

Individuals experiencing homelessness have a high prevalence of physical and mental illnesses. For example, in one community survey, 66% of homeless male veterans reported a chronic medical condition and 33% reported 2 or more mental health conditions.^5^ Homeless individuals also have an all-cause mortality rate that is 4.5 times higher than their housed counterparts.^6^ Moreover, homeless people face numerous barriers to care, including lack of health insurance, previous negative care experiences, transportation, limited availability of health services, stigma, lack of trust, care processes, and competing subsistence needs.^7-10^

Disasters can further exacerbate underlying access to care problems for vulnerable populations.^11-13^ Since 1970, natural disasters have led to the permanent closure of 5 VA Medical Centers (VAMCs) and the extended closure of 1 VAMC, disrupting inpatient, emergency, and outpatient services for thousands of veterans.^14-18^ High disease burden, shelter instability, social isolation, limited transportation, and lack of resources make homeless individuals especially vulnerable to adverse health effects of disasters.^19,20^ A recent study of emergency department utilization in New York City post–Hurricane Sandy found that homeless individuals were at increased risk for developing acute medical needs following a disaster.^21^ Facility closures, whether permanent or temporary, may disproportionately impact homeless VA patients who are more likely to experience increased adverse effects from disasters, yet simultaneously face limited access to care elsewhere.

Accordingly, we conducted a pilot study to examine the characteristics of homeless VA patients who reported that they would have difficulty obtaining care elsewhere if their the VA facility they most frequently visit was closed due to a natural disaster.

## Methods

We analyzed data from the VA Preparedness Communication Survey (VAPCS), which included separate probability samples of homeless and nonhomeless veterans in the Northeastern United States, an area affected by hurricanes and severe winter storms. This analysis concerns only the sample of homeless veterans. Analyses of the nonhomeless veteran sample are reported elsewhere.^22,23^

The sample frame for the study was drawn from the Veterans Health Information and Technology Architecture (VISTA) system via a data extract from the VA Corporate Data Warehouse (CDW) on May 29, 2015. Individuals were included in the sample frame if they (1) had an address in Veterans Integrated Service Networks (VISNs) 3, 4, and 5 (administrative service areas as defined in 2015, corresponding to the Northeastern United States) and (2) had at least 1 visit with the ICD-9-CM V60.0 diagnostic code “Lack of housing” or clinical documentation of having received specialized VA homeless services (stop code 528 = “Telephone/Homeless Mentally Ill” or 529 = “Health Care for Homeless Veterans [HCHV]”) within 24 months prior to the sample draw.^24^

Individuals who did not have a valid address and telephone number were excluded from the sample frame. Ninety-six percent had valid addresses and 91% of had valid phone numbers. Addresses included homes, apartments, PO boxes, group or transitional housing, VA hospitals, emergency shelters, or churches, reflecting the broad range of temporary living situations for homeless veterans. A random sample of 2000 was drawn from the 22 446 individuals in the sample frame who met eligibility criteria.

The survey contained 37 questions addressing topics related to accessing the VA during natural disasters, communication-related preferences during disasters, the types of disasters experienced, and disaster preparedness. This analysis examined questions related to difficulty accessing care during disasters. The survey was administered by a professional survey research company using a sequential mixed-mode method. Participants were mailed a cover letter and paper questionnaire, a web link to the survey, and $2 cash. The cover letter stated that respondents would receive an additional $20 cash on survey completion. Nonrespondents were called and asked to complete the survey via Computer-Assisted Telephone Interviewing, until a sufficiently large sample had been obtained. A total of 383 respondents completed the survey. The unadjusted and adjusted response rates were 19% and 20%, respectively. Responses were weighted using a standard 3-step approach applying a sample weight, nonresponse weight, and poststratification adjustment.

The dependent variable was based on the survey question: “Now think about the VA medical facility that you go to most frequently. If that VA facility was closed due to a natural disaster, how easy would it be for you to get care elsewhere?” Response categories included “very hard,” “somewhat hard,” “neither easy nor hard,” “somewhat easy,” and “very easy.” We used a conservative definition of difficulty accessing care during disasters where these response categories were dichotomized: difficult (“very hard”) versus not difficult (combining the remaining 4 response categories).

Univariate analyses were performed to describe the characteristics of the study population. Bivariate analyses using chi-square tests were conducted to examine the association between difficulty accessing care elsewhere during disasters and sociodemographic characteristics, health status, health insurance status, and frequency of VA visits. Multivariate logistic regression was performed to examine the association between difficulty accessing care during disasters and relevant study variables: age (<45, 45-64, >64 years), gender, frequency of VA visits (⩾5 visits to a VA health care facility in the prior 12 months), VA health care coverage, Medicaid, Medicare, private or other health insurance, mobility difficulty, and eyesight difficulty. All analyses were conducted using Stata (v.13 StataCorp, College Station, TX). Per the policies regarding activities that constitute research at the VA Greater Los Angeles Healthcare System, this study met criteria for quality improvement activities and was exempt from ethics review.

## Results

Overall, 23% of respondents (n = 87) reported that they would find it difficult to get health care elsewhere if the VA facility they frequented was closed from a natural disaster. Respondents in both groups, difficult (D) versus not difficult (ND) to access care elsewhere, were predominantly male (86.0%), unmarried (79.9%), had some college, a 2-year degree, or more education (56.9%), were racial/ethnic minorities (60.6%), were unemployed or disabled/unable to work (60.4%), and had a mean age of 52.8 years. Respondents in both groups reported functional health challenges: 48.8% reported difficulty concentrating, remembering, or making decisions; 29.2% reported poor eyesight; and 31.6% used equipment to assist with mobility. Respondents in both groups were frequent users of VA care, with 38.5% reporting 10 or more visits to a VA facility in the past 12 months (Table 1).

**Table 1.** Characteristics of Homeless VA Users in Northeastern United States by Difficulty Accessing Care During Natural Disasters.^a,b^

	Overall (%)	Difficult (%)	Not Difficult (%)
Age (years)			
<45	24.2	25.0	23.9
45-64	63.4	68.3	62.0
65+	12.4	6.8	14.1
Mean age, years (95% confidence interval)	52.8 (51.5-54.1)	51.6 (49.0-54.2)	53.1 (51.6-54.6)
Gender			
Male	86.0	82.5	87.0
Female	14.0	17.5	13.0
Race/Ethnicity			
White	39.4	41.8	38.7
Racial/Ethnic minority	60.6	58.2	61.3
Marital status			
Married/Living as married	20.1	20.0	20.2
Unmarried (divorced, separated, never married, widowed)	79.9	80.0	79.9
Education			
Less than high school graduate	5.1	3.0	5.7
High school graduate/GED or less	38.0	37.5	38.1
Some college or 2-year degree, or more	56.9	59.5	56.3
Employment status			
Employed	27.3	27.9	25.1
Unemployed	22.1	24.7	21.3
Unable to work or disabled	38.3	41.7	37.2
Retired	12.3	8.4	13.5
Difficulty concentrating, remembering, or making decisions			
No	51.2	48.3	52.1
Yes	48.8	51.7	47.9
Eyesight, without glasses or contact lenses*			
Fair/Good/Very good/Excellent	70.8	62.5	73.3
Poor	29.2	37.5	26.7
Use equipment to assist with mobility (wheelchair/cart/scooter, crutches, cane or walker, prosthetic or artificial limb)			
No	68.4	66.0	69.1
Yes	31.6	34.0	30.9
Number of VA health care visits in past 12 months			
<5	37.8	28.9	40.3
5-9	23.7	28.8	22.2
⩾10	38.5	42.3	37.4
Health insurance			
Has VA coverage	82.7	79.1	83.8
Has Medicaid coverage**	23.9	14.0	26.8
Has Medicare coverage	28.0	24.4	29.0
Has private or other insurance	16.6	11.3	18.2
n	383	87	296

a Source: VA Preparedness Communications Survey (VAPCS) (August-November 2015).

b Percentages may not total to 100 due to rounding error.

* *P* < .10. **P < .05.

Bivariate comparisons of the 2 groups found no statistically significant differences in sociodemographic factors, functional health status, or frequency of VA health care visits (Table 1). Although not statistically significant, a higher proportion of respondents with difficulty accessing care elsewhere reported poor eyesight (37.5% D vs 26.7% ND, *P* = 0.06). Medicaid coverage was significantly lower in the group reporting difficulty accessing care (14.0% D vs 26.8% ND, *P* < .05). Other forms of reported insurance coverage were similar across both groups.

In a multivariate logistic regression analysis predicting difficulty of accessing care during disasters, respondents with Medicaid coverage were less likely (OR 0.38; 95% CI: 0.18-0.78; *P* = .008) to report that they would have difficulty finding care elsewhere during future disaster-related VA facility closures. Although not statistically significant (at .05 level), respondents with VA insurance were less likely (OR 0.59; 95% CI: 0.32-2.09; *P* = .093) and respondents with eyesight difficulties were more likely (OR 1.61; 95% CI: 0.95-2.71; *P* = .076) to report that they would have difficulty finding care elsewhere during disasters (Table 2).

**Table 2.** Logistic Regression^a^—Difficult to Access Care Elsewhere if VA Facility Closed in a Future Natural Disaster.^b^

Variable	Reference Category	Odds Ratio	95% Confidence Interval	*P*
Age	Continuous	0.99	0.97-1.02	.472
Male	No (ref)	—	—	—
	Yes	0.67	0.28-1.58	.359
⩾5 visits to VA/12 months	No (ref)	—	—	—
	Yes	1.56	0.88-2.80	.131
Insurance_Medicaid**	No (ref)	—	—	—
	Yes	0.38	0.18-0.78	.008
Insurance_VA*	No (ref)			
	Yes	0.59	0.32-2.09	.093
Insurance_Medicare	No (ref)			
	Yes	0.77	0.41-1.43	.401
Insurance_Private/Other	No (ref)			
	Yes	0.58	0.27-1.27	.174
Mobility difficulty	No (ref)			
	Yes	1.22	0.71-2.12	.469
Eyesight difficulty*	No (ref)			
	Yes	1.61	0.95-2.71	.076
Constant		—	—	—
	—	0.75	0.20-2.8	.674

a Logistic regression using *svy* command in Stata to account for survey design; F (9, 368)=2.17, p=0.0237.

b Survey question: “Now think about the VA medical facility you go to most frequently. If that VA facility was closed due to a natural disaster, how easy would it be for you to get care elsewhere?” The 5 response categories were dichotomized: difficult (“very hard”) versus not difficult (Somewhat hard/Neither easy nor hard/Somewhat easy/Very easy).”

* *P* < .10. **P < .01.

## Discussion

Although access to care among homeless VA patients has been extensively studied,^8,25^ it has not been studied in disasters. Our findings suggest that having Medicaid may provide an important option for homeless VA patients to access care elsewhere if their primary VA facility is closed due to a natural disaster.

Beginning in 2014, the Patient Protection and Affordable Care Act (ACA) (Pub L No. 111-148; 42 U.S.C. §§ 18001-18121 (2010) gave states the option to expand Medicaid to adults up to age 65 years with incomes up to 138% of the federal poverty level. As of September 11, 2018, 34 states, including the District of Columbia, had adopted Medicaid expansion.^26^ Studies estimate a significant percentage of homeless VA users were likely eligible for Medicaid under the state Medicaid expansion option.^27,28^ While only 3% of homeless and at-risk veterans enrolled in VA health care were covered by Medicaid in 2014, 78% were likely eligible in states with Medicaid expansion.^27^

It is unclear how many homeless VA patients have enrolled in Medicaid. A recent study among uninsured homeless individuals found that the most common barrier to Medicaid enrollment was “not knowing if qualified for Medicaid,” followed by “not knowing the steps to take to sign up for health insurance.”^29^ Other factors such as language and literacy barriers, distrust of government organizations, and lack of transportation also pose barriers to enrollment.^30^ Given these challenges, homeless veterans may require substantial assistance with Medicaid enrollment. Some have suggested that VA homeless programs are well positioned to assist homeless veterans with Medicaid enrollment,^27^ although this would require VA staff to be trained and permitted to assist with eligibility for non-VA services.

Even though concerns have been raised that dual Medicaid-VA enrollment could lead to more fragmented care,^31^ lower quality of care, duplicative care, and higher costs,^28,32^ Medicaid coverage may offer critical access during disasters when VA medical facilities are closed or alternative VA medical facilities are limited. Natural disasters do not necessarily affect all healthcare facilities when they strike a community, and thus some non-VA facilities could be operational following a disaster even if all VA and some non-VA facilities close because of the event. Experience from Hurricanes Katrina and Sandy found that uninsured individuals and other vulnerable populations that had poor access to care in nondisaster periods also experienced compromised access following disasters.^11-13,33,34^ In contrast, low-income individuals with Medicaid coverage in an area directly impacted by a disaster had improved access to primary care in 24 months after the disaster.^35^ Our study suggests Medicaid may also improve access to care for homeless VA patients following disasters.

This study has limitations. The results cannot be generalized to homeless VA users nationwide or to states where Medicaid has not been expanded. Similarly, the results cannot be extended to homeless veterans who do not use VA services or nonveteran homeless populations. While most studies of homeless veterans have relied on convenience samples, we used a population-based sampling strategy relying on mailing addresses and phone numbers as the inclusion criteria for the sampling frame. VA patients, including homeless patients, have a strong incentive to maintain up-to-date contact information, even if temporary, on file to receive VA benefits. In this study, less than 1% (n = 128) of the surveys were returned to the sender by US Postal Service. However, this approach may have biased the sample toward sheltered homeless VA users (eg, those in transitional housing, longer term shelters, VA domiciliary) and those who are able to visit a PO box or other mailing address on a regular basis. As a result, some of the most vulnerable members of the homeless population might not have been represented. Although the response rate for this survey (20%, adjusted) is relatively low, it reflects the challenges of conducting population-based surveys with hard-to-reach populations.^36^ Increasing the response rate in surveys of hard-to-reach patient populations is a difficult, but worthwhile, objective for future studies to increase power. In addition, it is conceivable that lack of sufficient power and additional factors or variables not included in the survey may have accounted for nonsignificant findings. Finally, all data were self-reported.

Future research should examine the extent of dual VA-Medicaid enrollment among homeless VA patients, and whether those individuals have used Medicaid in lieu of VA care during disasters. In addition, there is value in determining whether results from this study would generalize to other Medicaid expansion states with a sizable homeless veteran population (eg, California), and examining whether homeless veterans without Medicaid are at greatest risk for adverse health consequences during disasters.

## Conclusions

Having Medicaid coverage may provide an important option for homeless VA patients to access care elsewhere during disasters. Although it is unclear how many homeless VA patients are dually enrolled in Medicaid, policy changes (eg, block grants, per capita caps, work requirements) that affect Medicaid eligibility or coverage^37,38^ could limit access to care for homeless veterans when VA health facilities are closed because of disasters.
